# Correspondence

**Published:** 1884-07

**Authors:** G. Fleming


*To the Editors of the Journal of Comparative Medicine and Surgery:*

*Gentlemen:* In your issue for last April there is an article on "Eclampsia Parturientum," which is alleged to have been read before the Gynecological Society of Boston by Mr. F. S. Billings. In that article I find the following passage: "For Veterinary medicine, it is sufficient to say that Franck, the best authority on Animal Obstetrics, has adopted and very ably defended it. Mr. Fleming's work on the same subject is but a literal translation of Franck's."

Will you kindly permit me to assert that this statement of Mr. Billings is absolutely and wholly untrue. My work on Veterinary Obstetrics is in no respect a translation of that by Franck, as any one may readily see who will take the trouble to compare the two books. Neither have I in any way made a mistranslation of anything Franck has written. There are other books on the subject besides Franck's, and which were published long before his. . . Yours obediently,

G. Fleming.

